# The Factors Influencing the Sense of Home in Nursing Homes: A Systematic Review from the Perspective of Residents

**DOI:** 10.1155/2016/6143645

**Published:** 2016-05-23

**Authors:** M. D. Rijnaard, J. van Hoof, B. M. Janssen, H. Verbeek, W. Pocornie, A. Eijkelenboom, H. C. Beerens, S. L. Molony, E. J. M. Wouters

**Affiliations:** ^1^Fontys University of Applied Sciences, Institute of Allied Health Professions, Dominee Theodor Fliednerstraat 2, 5631 BN Eindhoven, Netherlands; ^2^Fontys University of Applied Sciences, Fontys EGT-Centre for Health Care and Technology, Dominee Theodor Fliednerstraat 2, 5631 BN Eindhoven, Netherlands; ^3^Fontys University of Applied Sciences, Fontys School of People and Health Studies, Dominee Theodor Fliednerstraat 2, 5631 BN Eindhoven, Netherlands; ^4^CAPHRI School for Public Health and Primary Care, Department of Health Services Research, Maastricht University, Duboisdomein 30, 6229 GT Maastricht, Netherlands; ^5^EGM Architecten, Wilgenbos 20, 3311 JX Dordrecht, Netherlands; ^6^Quinnipiac University School of Nursing, North Haven Campus, Office MNH 470P, 275 Mount Carmel Avenue, Hamden, CT 06518-1908, USA

## Abstract

*Purpose*. To provide an overview of factors influencing the sense of home of older adults residing in the nursing home.* Methods*. A systematic review was conducted. Inclusion criteria were (1) original and peer-reviewed research, (2) qualitative, quantitative, or mixed methods research, (3) research about nursing home residents (or similar type of housing), and (4) research on the sense of home, meaning of home, at-homeness, or homelikeness.* Results*. Seventeen mainly qualitative articles were included. The sense of home of nursing home residents is influenced by 15 factors, divided into three themes: (1) psychological factors (sense of acknowledgement, preservation of one's habits and values, autonomy and control, and coping); (2) social factors (interaction and relationship with staff, residents, family and friends, and pets) and activities; and (3) the built environment (private space and (quasi-)public space, personal belongings, technology, look and feel, and the outdoors and location).* Conclusions*. The sense of home is influenced by numerous factors related to the psychology of the residents and the social and built environmental contexts. Further research is needed to determine if and how the identified factors are interrelated, if perspectives of various stakeholders involved differ, and how the factors can be improved in practice.

## 1. Introduction

For older people who can no longer age in place, nursing homes provide an alternative place of residence where care and assistance are offered by professionals [1]. Nursing home residents no longer live in their “own home.” Because a nursing home has a dual nature as an institution and as a home, many health care organizations try to provide living arrangements that focus on “the good life” and the creation of an environment that is like a home to its residents, instead of a health care facility in which they reside [2, 3]. At the same time, the prevalence of dementia and care dependency in nursing homes increases (e.g., Schüssler et al. [4]). However, delivering both good (clinical) care and a homelike environment is challenging. In the near future, the demand for care of nursing home residents will increase as residents deal with the effects of dementia or severe physical limitations. At the same time, there is a trend towards small-scaled and homelike accommodations. To date, the exact elements that shape the physical, social, and organization contexts are largely unknown.

Health care organizations across The Netherlands are responsible for providing the basic furnishing of a room within a nursing home. With all basic clinical requirements a room should fulfill [5–7], the question about how to provide residents of nursing homes with a sense and feeling of home remains. Admission to a nursing home is a major life event, as most individuals do not wish to leave the home they have been living in for a long time in order to move to a nursing home [8]. Nevertheless, “*there seem to be good reasons to assert that living in an institution and being 'at home' is not a contradiction in terms*” [9, p. 221].

In the second half of the 20th century, the quality of the nursing home building was often expressed in terms of technological, functional, and economic requirements [10]. Hence, requirements related to the personal preferences of the residents were given less attention. A more holistic vision of health care is currently emerging and considers the consequences of the built environment on the well-being of the residents [10]. For instance, Van Steenwinkel et al. [10] conducted a study focusing on how the built environment contributes to a sense of home of older people living in different contexts in Belgium. Van Steenwinkel et al. [11] investigated the sense of home among permanent and temporary residents of nursing homes in The Netherlands. Both studies concluded that a sense of home is a multifactorial phenomenon which is highly influenced not only by the (built) environment, but also by social and personal characteristics. A sense of home is related to personal experiences and emotions and does not come into existence overnight but is gradually developed by the person in whom independence, security, and the source of own identity, choice, and controls, as well as memories, are essential [12–16]. Fully understanding the experienced sense of home of nursing home residents from both the insider and a holistic perspective might have important implications for the development of policy of health care organizations and the innovations in nursing homes of the future [17]. Conceptual frameworks and constructs concerning a sense of home that have been developed and researched from these perspectives are assumed to be relevant to this effort. According to Duyvendak [18], there are two situations that support the process of feeling at home: “haven” (a place that is secure, comfortable, and predictable; a place where one can feel at ease) and “heaven” (a place where you can be who you are and feel connected with like-minded people) [18].

The objective of this study is to systematically review which factors contribute to a sense of home for older people living in residential care facilities. To date, a list of specific factors contributing to the sense of home experienced by nursing home residents is not yet available to nursing home organizations, family members, architects, and other stakeholders. An understanding of these factors is necessary to build and test hypotheses that may predict and eventually improve experiences of home in residential care facilities.

## 2. Methodology

### 2.1. Search Strategy

A systematic literature search was performed in May and June 2015. In order to scan the literature for factors which contribute to the sense of home for older people living in nursing homes, five databases (CINAHL, PsycINFO, PubMed, Scopus, and Web of Science) were searched using a combination of two groups of keywords: (1) “meaning of home”, “sense of home”, and synonyms for these terms; (2) “care home” OR “nursing home” and similar search terms. Truncation was used when variations of the used nouns were commonly used in the literature. The full list was (“at-homeness” OR “meaning of home” OR “sense of home” OR “feeling of home” OR homelike OR homeliness or “experience of home”) AND (“care  home^*∗*^” OR “nursing home^*∗*^” OR “institutional care” OR “long-term care” OR “long term care” OR “care institution^*∗*^” OR “residential care” OR “residential home^*∗*^” OR “assisted living” OR “small scale setting^*∗*^” OR “small scale living” OR “small scale facilit^*∗*^” OR “shared housing arrangement^*∗*^” OR “special care facilit^*∗*^” OR “institutionali^*∗*^” OR “sheltered housing” OR “small scale group accommodation^*∗*^” OR “elderly hous^*∗*^” OR “elderly home^*∗*^” OR “special care unit”). Furthermore, references of the included articles were checked for additional articles eligible for this review (snowball method).

### 2.2. Article Selection

Titles, abstracts, and full articles were subsequently screened by the principal investigator [M. D. Rijnaard] for the inclusion criteria mentioned in the following list. In case of doubt, three authors [M. D. Rijnaard, E. J. M. Wouters, and J. van Hoof] discussed the selection first and consulted the project group if consensus was not reached.

*Inclusion and Exclusion Criteria*

 Inclusion criteria include the following:
   (i) Original and peer-reviewed articles written in English.
   (ii) Data collected from residents living in nursing homes and other stakeholders.
   (iii) Aiming at investigating factors that influence the sense of home, meaning of home, at-homeness, or homelikeness.
   (iv) Data collected from studies which aimed to find out what contributes to a sense of home for the residents through the eyes of residents, family, or care staff.
   (v) Being qualitative, quantitative, and mixed-method studies.
 Exclusion criteria include the following:
   (i) Short stay in nursing home.
   (ii) Assisted living or congregate living.
   (iii) Studies not written in English.

*Note*

- No exclusion criterion is based on country, continent, age of the study, residents' age, and year of publication.

### 2.3. Data Extraction and Analysis

Thematic synthesis was used to synthesize data into factors. Prior to the literature search, a meeting was held during which the strategy of the literature search was set out. Choices were made about which databases to screen, which key terms to use, and what inclusion and exclusion criteria would be.

One author [M. D. Rijnaard] read all articles and extracted data which described a relationship with sense of home. Four other authors [J. van Hoof, B. M. Janssen, E. J. M. Wouters, and H. Verbeek] each read part of the papers and also extracted data, which described a relationship with sense of home. Next, M. D. Rijnaard paired up with each of the four authors mentioned before, and they together reached consensus on the extracted data, that is, open codes. The data extraction identified a large number of quotes which are used in the Results section as a presentation of the data to exemplify the meaning of a specific factor. After this open coding, multiple sessions were held to group factors derived from the reviewed articles (axial coding). The first meeting of axial coding involved four authors [M. D. Rijnaard, B. M. Janssen, W. Pocornie, and H. Verbeek], and three subsequent meetings each involved two authors [M. D. Rijnaard and J. van Hoof; M. D. Rijnaard and W. Pocornie]. Additionally, several sessions were held [M. D. Rijnaard and J. van Hoof] and an additional session was held [M. D. Rijnaard, J. van Hoof, and W. Pocornie] to create a conceptual model as an expression of the thematic synthesis, which shows the relationships between themes.

## 3. Results

**Figure 1:**
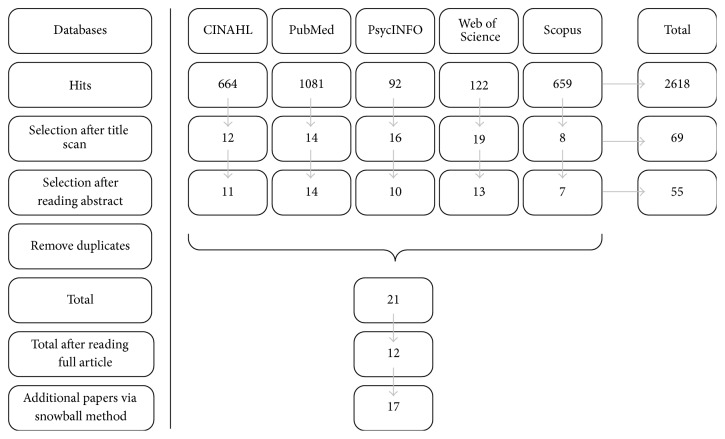
Flow diagram of the article selection process.

**Figure 2:**
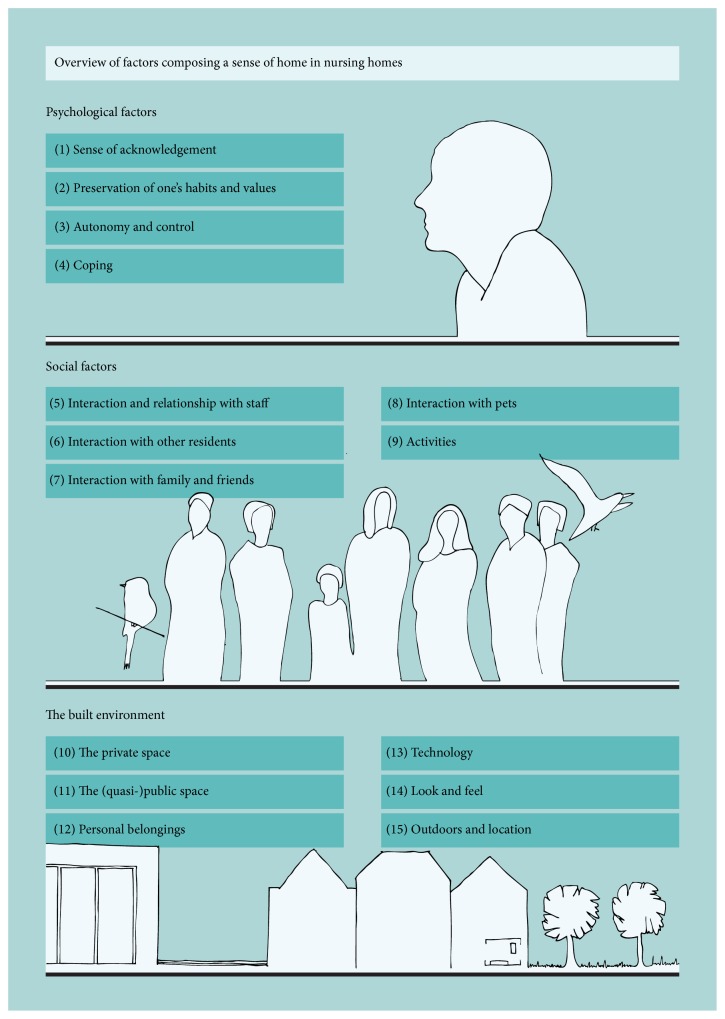
Overview of factors composing a sense of home in nursing homes.

The search in five databases generated a total of 2,618 results (Figure 1). The selection process initially led to the inclusion of 21 articles. After reading the full text, 12 articles remained. The snowball method added five articles, bringing the total number of articles included in this review to 17. The characteristics of these studies are described in Table 1. Of these studies, six were conducted in The Netherlands, four in USA, two each in Sweden and Norway, and one each in New Zealand, Ireland, and Australia. From these studies, 15 factors were identified that impact the sense of home of nursing home residents (Figure 2). Some of these factors have a certain degree of overlap, as boundaries are not sharp but gradually blend into each other. As a consequence of the process of clustering of factors, three main themes emerged as follows:

- Psychological aspects, which include behavioral, cognitive, and emotional components.
- Social aspects, which include home as a place of connection and socialization.
- The built environment, which includes the layout of a space, its interior design, and the surroundings.

### 3.1. Theme: Psychological Factors

Among the factors constituting the psychological aspects of the sense of home are the sense of acknowledgement, the preservation of one's habits and values, autonomy, and control, and coping.

#### 3.1.1. Sense of Acknowledgement

There are numerous factors within the theme of sense of acknowledgement that contribute to a sense of home among nursing home residents. Molony et al. [27] identified a relationship with staff who “really cared” and having fun with staff and other residents among the relevant factors associated with at-homeness. Fun and friendship and laughing with one another are important too. Robinson et al. [29, pp. 498-499] stated that “*knowing the person ‘underneath' the dementia*” and “*enhanced family access to staff as regular conversations occurred in the midst of everyday activities*” are important for the creation of a sense of home in small-scaled group accommodations for people with dementia. de Veer and Kerkstra [20] also underlined the need for resident-centered attitudes of staff. These positive interactions with staff members and other residents were also mentioned by Lewinson et al. [26, pp. 750-751]. Having things in common with other residents contributes to establishing new relationships [12]. Feeling respected and feeling known by the people in a facility make it a home [26, p. 751]. In addition, the style of working of care professionals has an influence [25, p. 96].

*What the caregivers did change was their style of working. They provided the residents with more control and choice within the daily routines by listening more carefully and by consulting the residents.*

Cooney [15, p. 192] also stressed the aspects of feeling known and valued as an individual. The identity of residents was strongest when their sense of their own value and uniqueness and other people's (particularly staff) actions that communicated their worth matched.

#### 3.1.2. Preservation of One's Habits and Values

Engaging in domestic chores and trying to live in the same manner as one always had were experienced as moments of privacy imperative for feelings of being at home [21, p. 1002]. Within the private sphere, residents should be able to engage in domestic chores. Nakrem et al. [28] added that women in particular felt that their role had been uncomfortably altered when admitted in a nursing home, as the engagement in old habits was impaired. There seemed to be a tension between the necessary institutional routines and the residents' personal habits, which resident should be able to continue. Nakrem et al. [28] mentioned the positive effects of support in self-activation which could make a great difference to the resident, by exploiting one's own strengths.

In addition, Robinson et al. [29, p. 500] found that there were competing demands for staff who were charged with creating a “homely atmosphere” in nursing home cottages, in which they had to choose between carrying out household activities and addressing serious health care needs. Through respecting the residents' wishes, the caregivers provide room for residents' self-determination [25, p. 97]. Continuity in activities from one's previous home-life is important too [25]. Items related to practicing a hobby are important even though many of the hobby activities were too complicated to carry out in the nursing home, because of impairments and limitations [16]. Cooney [15, p. 191] found that the continuity in normal activities and day-to-day activities and rituals are important for experiencing a sense of home. This is illustrated by a quote about the route of a facility by Cooney [15, p. 192].

*What was noticeable was that more residents with an ‘inward gaze' resided in settings where the [*clinical*] routine was dominant.*

Cooney [15, p. 191] stated that the degree to which participants could maintain personal routines mirrored the degree to which they were able to exert control over their day-to-day life. Carboni [19], Nakrem et al. [28], and Bland [12] stressed the negative impact of routines, rules, lack of control, and intrusions to privacy. Cooney [15, p. 193] continued by stating that residents' life experiences, values, and expectations of long-term care shaped both their initial response to long-term care and their long-term experience. Fleming et al. [22, p. 6] further added the engagement with one's senses (sounds, sunshine, and scents) and spiritual engagement as a way of nurturing one's core self to the aforementioned factors. Fleming et al. [22] also concluded that it is important to respect a person's dignity while carrying out care and that it is important for care professionals to see residents as unique individuals.

#### 3.1.3. Autonomy and Control

Autonomy and control are frequently mentioned factors in relation to having a sense of home in a nursing home environment [2, 16, 29, 30]. van Dijck-Heinen et al. [2, p. 65] described autonomy as being able to do what you want to do, even though not everyone will develop a sense of home when given full autonomy.

*You feel like home when you can do whatever you want to do. Grab your own belongings, or a book whenever you want to read. Or play music when you want to listen to music. Or paint a painting when you want to.*

Perceptions of freedom and mobility were identified by Molony et al. [27, p. 510] as ingredients for high levels of at-homeness. Privacy, being able to do things for oneself, and being able to care for oneself were considered to be important factors by residents too [27, p. 511]. van Hoof et al. [30] also identified the freedom of placement as important, for instance, having access to a taxi service. Lewinson et al. [26, p. 752] found that scheduling transportation was a problem to residents. Residents described that as “feeling trapped in the facility.” In the study by van Hoof et al. [16], some of the residents were unsure whether they were allowed to stay in the present room or whether they needed to move within the nursing home. These participants said that they were unsure whether they would ever develop a sense of home.

In relation to decorating their own rooms, residents should be allowed to hang things on the walls and be allowed to drill holes [16]. According to Falk et al. [21], being in charge is a dimension of attachment to place. Being allowed to independently decide whom to include and to exclude is important too. Inviting a coresident to one's private room is equivalent to that of inviting someone into one's home [21]. Residents should have the ability to find pride in managing even the smallest chores by themselves.

*George's private bedroom in the rest home wing provided him with little sense of sanctuary, especially as he was still unable to prevent other residents entering at will. [12, p. 7]*

Cooney [15, pp. 192-193] continued by stating that participants who “created” their own space usually considered the facility their home. Their comments suggested a feeling of “ownership” of their space.

Whether participants' move to long-term care was voluntary or involuntary was a determining factor in whether they found a home or not [12, 15]. Klaassens and Meijering [25] found that residents should be allowed to do as they wished, and there should be a shift away from risk avoidance to risk management. Being able to lock one's own door improves the sense of control and not “being locked up.” In the study by van Dijck-Heinen et al. [2], the rules of nursing homes and the practices of care professionals were considered to be too steering. Shared decision-making, for instance, about medication regime, can alleviate this situation. Group living can lead to forced and sometimes unwanted relationships breaching the sense of autonomy of residents. In line with this factor, pieces of furniture that were easily moved provided residents with enhanced self-determination [24].

#### 3.1.4. Coping

The fourth psychological factor is coping. First of all, the nursing home placement itself is a serious life event. It may lead to suffering and grieving losses or feelings of guilt for being unable to prevent placement, second guessing themselves with regard to the necessity of placement, and concern over the circumstances of care [29, p. 496]. Connectedness to the future appeared to be entirely severe as many individuals spoke with resignation of dying in the nursing home [19, p. 35]. In the study by van Hoof et al. [30, p. 6], participants stated that “*living in the nursing home will never be like living at home but that they have accepted the situation and are satisfied.*” In the study by van Zadelhoff et al. [31, p. 2493], residents were settled, at ease, restful, and accepting the situation and life as it was. Nakrem et al. [28, p. 220] see the acceptance of being together with people with problematic behavior as a strategy to cope with living in the nursing home.

Residents may use various strategies to cope with the move to long-term care, with strategies as “not looking back,” “making yourself happy,” and “being good” [15]. If residents are unable to cope, they may actively refrain from personalizing the private room, which strengthens the notion that home was someplace else for them [21]. One may either accept or reject being frail, look on the bright side of life, or give up and feel discarded [21]. In addition, some residents are ashamed of their “corporeal decay,” which in turn contributes to isolation and strengthens feelings of being an outsider. Negative self-conceptions about being a burden to others, including staff, can also hamper a sense of home [21, 28]. Part of the coping may be emphasizing the advantages of living in a nursing home, such as safety and receiving prompt emergency help as the main advantages [28].

### 3.2. Theme: Social Factors

Among the factors constituting the social aspects of the sense of home are the interaction and relationship with staff, fellow residents, family and friends, pets, and activities. The study by Cooney [15, p. 192] described that participants associated a feeling of “belonging” with feeling at home. “Belonging” was defined as being part of the group and experienced as a sense of solidarity, companionship, relaxation, and fun. Carboni [19, p. 34] further added that connectedness with people was severely limited or completely lost in a nursing home. Falk et al. [21, p. 1003] found that socializing with others made residents aware of their physical appearance, and the opportunity to dress up and to have one's hair set was important to maintain continuity of sense of self-identity and personal values. In the following section, these relationships, interaction, and activities are described in greater detail.

#### 3.2.1. Interaction and Relationship with Staff

The most important category concerning interaction is that with members of staff, that is, the professional caregivers residents see and interact with on a daily basis.

Close interpersonal relationships with members of the staff who “really cared” are associated with at-homeness [27]. The affectionate manner that residents are being cared for contributes to a sense of home [30].

van Dijck-Heinen et al. [2] explicitly stated that a disrespectful treatment by a member of staff and not being able to choose your own friends were hindrances to a sense of home. Participants also indicated that caregivers do not always live up to promises. Still, they preferred receiving help from a care professional instead of via technological solutions. A caregiver is a point of contact and provides a sense of privacy, safety, and security [2].

Miscommunication was also mentioned by Lewinson et al. [26] as a source of not feeling at home. An example of poor communication between staff and residents was slipping informational sheets under resident doors [26, p. 752] as a way to communicate. The use of language of practitioners was studied by Fleming et al. [22, p. 9]. The practitioners' use of language revealed embeddedness within institutional systems and processes and this was particularly evident when they referred to patients. Falk et al. [21, p. 1005] also wrote that the language sometimes used by the staff connoted the powerlessness of residents that was related to feeling dismissed. Moreover, respondents lacking trust in nursing staff felt reluctant to speak freely when they thought something was wrong. A fear of reprisals undermined their sense of self-worth and dignity.

The literature revealed several do's and don'ts related to staff interaction. The social environment is a mix of residents' interactions with each other and with staff, which is influenced by wider issues, for example, staff values and practices [15, p. 194]. Klaassens and Meijering [25] found that that the presence of a care professional had an important effect on the atmosphere in the communal room and as a result of some simple measures. Many residents liked to talk to and spend time with the caregivers. As staff in this study did not wear a uniform, there was no visual distance. Interactions reshaped the relationships between caregivers and residents, closing the gap between them and balancing power relations, making them more equal parties. To increase residents' participation in decisions about their treatment and care, residents were allowed to take part in the multidisciplinary meetings and to visit the nursing home doctor themselves [25]. In the study by Falk et al. [21, p. 1003], doing activities with staff was mentioned. For instance, sharing autobiographical memories and old photographs, to talk about day-to-day events and in-home, were important to maintain a sense of continuity of self-connecting with the past, as well as becoming acquainted and getting to know each other.

In the study by Nakrem et al. [28, p. 222], the residents referred to the staff as “kind,” “pleasant,” and “clever,” and many thought that the staff cared for them on a personal level. At the same time though, staff did not know the residents' names and staff felt that they did not have the energy to get to know them. Moreover, residents said that it was strenuous to repeatedly explain to new caregivers how to do the procedures. The quality of the staff was judged by their interest or motivation for doing something extra for the residents. Some nurses only did tasks that were expected of them and they were not able to give emotional support. In the study by Hauge and Heggen [23, p. 464], the “*residents were eager to answer questions and to discuss everyday matters with the staff. This sometimes ended up in cheerful discussions with laughter and smiles – what we have called golden moments.*”

In order to experience these “golden moments,” there are numerous aspects related to staff that need to be fulfilled. According to Robinson et al. [29, p. 497], a lack of adequate staff to provide attentive, individualized care is detrimental to the sense of home. Nursing homes may become “more like a hospital,” and staff may be overloaded in terms of the number of residents they care for. In the same study, also family participants noticed that the quality of the home was significantly influenced by the workload, the personality and strengths of the professional caregiver, and the fit between the resident and the setting of care. In the study by Nakrem et al. [28, p. 221], the residents thought that the staff were too busy to be able to provide attentive care.

*Many felt and that they could not ask for more help because there was always someone else that needed to be prioritized. [28, p. 221]*

This notion is also shared by practitioners, who were concerned about the lack of staff to provide sufficient care to their residents [22, p. 9] or who indicated that they would like to pay more attention to each individual resident [31, p. 2495]. In the latter study, conducted in small scale group accommodation, staff also indicated that it is important to know how the residents used to live and their personal biography. In this way, staff have a better understanding of residents' behavior and this improves the interaction. Staff are more perceptive and talk more about their private life than in a regular setting. They spend a part of their free time organizing and joining activities, which makes the connection with residents more intimate than in a regular setting [31, p. 2496]. Falk et al. [21] concluded that the dignity of the residents was violated when nursing staff made them wait for help or ignored their wishes. On this topic, Cooney [15, p. 194] made a remark at the level of individual staff members; three key factors impacted on residents' experience, that is, the “availability,” “reliability,” and “flexibility” of staff. It was important to participants that staff were physically and emotionally accessible to them.

Therefore, ideally, staff should be able to balance stability and predictability with flexible routines that matched the individual needs of the resident [29, p. 498].

Apart from issues related to the organization of the work, there are also some personal staff characteristics believed to contribute to relational connection with the residents, such as well-developed observation skills, personal interest in each resident, a sense of humor, ability to take things in their stride, attention to details, caring, kindness, and respect for the residents as persons [29, p. 498]. The individual staff member needs to be multitalented, have time to devote to their relationships with both residents and families, and be able to make informed decisions about the most effective manner of delivering individualized care [29, p. 504]. de Veer and Kerkstra [20, p. 432] stated that showing interest in the resident, providing a quick response to a request for help, and a friendly attitude were important.

Apart from the direct relationship between staff and residents, also the staff's attention for family is of great value. This is confirmed in studies by Robinson et al. [29, p. 500], which highlights the importance of a family-centered approach to care where there is strong recognition that illness is a family affair even when the ill member resides outside the family home. In the study by van Zadelhoff et al. [31, p. 2495], it was found that family feels that nursing staff can spend more time with individual residents in a relaxed way. Robinson et al. [29, p. 498] stated that staff may become acknowledged as the residents' second families. The “real relatives” believed it was a staff responsibility to take a stand and be firm in inviting, encouraging, or leading the resident to engage in healthful daily activities. While supporting the belief that residents should not be forced, they did not believe the only alternative was letting the resident do what they preferred when it was clearly not in their best interest [29, p. 503]. Transparent communication between staff and relatives, therefore, is of great importance to balance mutual expectations.

An agreement on the use of keys also appears to be important in the relationship and respect of privacy and dignity between staff and residents. Although the nursing staff had keys in the study by Falk et al. [21, p. 1003], the locked door symbolized privacy. The staff was welcome when the respondents needed assistance. A locked door symbolized that no one was welcome to enter, and a breach of that rule was experienced as infringement.

Nakrem et al. [28, p. 221] raised the issue of the interaction with staff during mealtimes. Residents were disappointed that the consumption and quality of the meals and food were “institutional” and not homelike. As the staff did not have time to sit down with the residents during meals, they served ready-made sandwiches to save time. However, the residents felt they could not help themselves in the unit kitchen, mainly because the area was perceived as controlled by the staff. In the study by Klaassens and Meijering [25, p. 97], staff would also eat or drink coffee with the residents, besides cooking, which highly contributed to the homelike feeling. Therefore, using meals together seems to be important.

Reciprocity in care relationships is an important aspect of relating with staff. Bland [12, p. 8] found that there was no role for residents, other than being the passive and grateful recipients of care. Experiencing symmetric power relations to the nursing staff was an important dimension of feeling valued and was experienced as reliant and well inclined, which contributed to sense of self-worth [21, p. 1004]. This could be organized through having respondents to join homelike activities, such as setting the table and folding linen [31]. Residents could help in the preparation of meals in the kitchen. This creates domestic circumstances and feelings, and residents make a positive contribution. Lewinson et al. [26, p. 752] stated that residents believed they should be involved by sharing their unique talents and hobbies with other residents.

#### 3.2.2. Interaction with Other Residents

Social relationships with and between fellow residents are very important to feeling at home in the nursing home [2, 27]. In the study by van Hoof et al. [30], a number of residents valued the dining atmosphere, especially in relation to being seated around a table together. van Zadelhoff et al. [31] found that residents are gathered together in the living room during the day. They talk with one another, drink coffee, or read a magazine. According to Cooney [15], the social environment was a mix of residents' interactions with each other and with staff. Klaassens and Meijering [25], too, stated that the residents shared their daily life with other residents. The participants in their study typically described their fellow residents as acquaintances rather than friends, though a small number of residents said that there were some fellow residents with whom they got along. The researchers' observations showed that not much interaction occurred between the residents during their study. de Veer and Kerkstra [20] also found that residents may feel disturbed, instead of being friends with, by other residents. Nakrem et al. [28] stated that spending time with the other residents was both an opportunity to be socially active and a source of irritation. In this study, an unbalanced mix of gender in the unit had made social relationships more superficial and some residents experienced that differences in interests sometimes resulted in disagreements. For instance, being the only cognitively intact resident in a unit could make it impossible to talk with the other residents [12, p. 7].

#### 3.2.3. Interaction with Family and Friends

According to Molony et al. [27, p. 509], closeness and involvement with family are associated with at-homeness in a nursing home environment. In the study by van Hoof et al. [30, p. 6], a number of participants indicated that they valued their contact with relatives as most positive, in particular having close contacts and being visited on a regular basis. Robinson et al. [29, p. 496] found that family members were considered the best caregivers for persons with dementia. Both Lewinson et al. [26] and Bland [12] spoke of the importance of visits by friends and relatives, who should be incorporated into planned activities, and of occasional visits back home. Both van Dijck-Heinen et al. [2] and Bland [12] made remarks about living together with a spouse. In the Dutch study [2], the residents stated that it should be possible to move into a nursing home with a partner. In the study from New Zealand, having to live apart from a spouse was a source of concern. van Zadelhoff et al. [31, pp. 2494-2495] found that relatives felt more involved in the group life in the context of small scale nursing homes than in a traditional nursing home. In small scale nursing homes, relatives were treated as group members instead of visitors. Furthermore, family frequently joined dinner and drinks. Despite their importance for the well-being of residents, relatives sometimes experienced tensions concerning the expected responsibilities and about their roles in relation to staff members [31].

#### 3.2.4. Interaction with Pets

Interaction with pets may complement the interaction with other human beings and may be a great source of distraction and joy during the day. Pets provide opportunities to engage when other people are not available. van Hoof et al. [30], for instance, found in their study that in one of the living rooms in a gerontopsychiatric ward there was a wild crow, eating from a net of peanuts hanging in front of the window. Residents considered these wild birds as pets. An animal gives a sense of consolation and warmth and makes a resident feel less lonely [2]. In the study by Fleming et al. [22], social engagement was raised by several participants and included engaging pets and even dolls.

#### 3.2.5. Activities

Activities are essential to make it through the day and provide meaning to life. Activities can be conducted alone or with members of staff, relatives, fellow residents, and others. Some residents engaged in activities arranged by the community, which help them preserve their continuity with the past and society, as well as reaching beyond the institution [21, p. 1004]. Lewinson et al. [26] found that the inability to get out of the facility more frequently to enjoy a variety of community venues was a common complaint. At the facility itself, residents were able to engage in social activities that included bingo, group exercise classes, cookouts, book club discussions, and other planned events. But at the same time, residents reported that activity planning problems inhibited feeling at home. Other activities mentioned by van Hoof et al. [30] were a cooking club, arts and crafts such as paint workshops, and reminiscence activities conducted in the activity space. Several studies found that activities and stimulation should be appropriate to the residents' needs, skills, education level, and abilities, including the need for customized activities or more private activities with relatives [2, 15, 22]. Outings, for instance, with spouses, girlfriends, or children, are also important activities [2, 25, 27]. In the case of small scale group accommodation [25, 29, 31], scheduled activities have been incorporated into daily life, which enhanced satisfaction and evoked a sense of homeliness. Residents actively engaged in meaningful activities, for instance, domestic tasks such as cooking. Participants who for some reason were unable to participate or who found that the activities did not suit them were forced to fall back on their own resources [15, p. 192], having to organize their own activities and not being able to join in group activities.

### 3.3. Theme: The Built Environment

Factors constituting the theme built environment include the private space and the (quasi-)public space, personal belongings, technology, look and feel, and the outdoors and location.

#### 3.3.1. The Private Space

The private space, whether it is a single-person room or a room shared with other residents, has an impact on the sense of home. In the study by Nakrem et al. [28, p. 220], “home” was associated with having a private room in the nursing home. A shared bedroom was unacceptable for most residents in this study. The desire for a private room may have a foundation in having opportunities to be on one's own, wishing for privacy, and having personal belongings around oneself [15, p. 192]. Central to nesting and the creation of attachment to place was spending time in one's room or apartment and being engaged in domestic chores [21]. Both findings by Klaassens and Meijering [25] and de Veer and Kerkstra [20] stressed the need for being on your own and withdraw to the own room, the opportunity to create your own environment. The need for privacy seems to be a main driver for having a private room. Private rooms govern the opportunity to talk with others in private or to withdraw from the communal living areas [16, 20, p. 432, 25]. The spontaneous opening of doors of private rooms is one of the actions hindering having privacy [2]. In this study, residents indicated that having a private room with private sanitary facilities was a must reported by all participants. Another desire in this study was to have a separate bedroom or recess for sleeping [2, 16].

Residents also indicated that having social interaction with the neighbors or coresidents should be facilitated through the design of the building [2]. Some of the residents asked for more spacious rooms, in particular the shower and kitchen areas. Also many residents in the study by Jonsson et al. [24, p. 28] felt that their private rooms were overfurnished and they wanted a larger space. van Hoof et al. [16] found that residents with larger apartments indicated that they could have visitors when they wanted. There was also the wish for having multiple rooms, a spare room that can be used when guests are staying for the night, a room for tools, or a work corner with a desk. In contrast, Klaassens and Meijering [25] found that the majority of the residents had no problem with the small size of the room.

Sanitary facilities are often mentioned as being important for residents, their comfort, and their feeling at home. The study by Robinson et al. [29] found that a private space including large private bathrooms with showers impeded a sense of home, because showers were unused as tub bathing was preferred by most residents. The private bathroom is also mentioned in other studies [22, 25, 30]. The bathroom can be a source of joy, if people can access it without difficulties, and they can wash and shower themselves. When having to share a bathroom with another resident, this may result in a lack of control around the use of the toilet. In the study by Fleming et al. [22], there were some differences in opinion on the necessity of ensuring privacy through having an ensuite or shared bathroom and a single bedroom or shared bedroom.

#### 3.3.2. The (Quasi-)Public Space

Many recommendations were made regarding the design of the public space, that is, the indoor space that resident shares with others. As some residents claim sections of this public space as their own, in this paper we describe this set of factors as quasi-public space. Hauge and Heggen [23], for instance, stated that the living room reflected unclear and somewhat inconsistent expectations. The boundaries between the public and private spheres are ambiguous and thereby differ from the comparatively sharp boundaries characterizing a home.

In general, smaller residential density, including family-style dining, increased perceptions of belonging [27, p. 512]. In many facilities, residents may have their own place around the (dining) table [30, p. 6]. The spatial layout in shared spaces with lounge furniture and dining tables may create an inviting atmosphere to share a cup of coffee and some talk with nursing staff and coresidents [21, p. 1003]. Also the study of Fleming et al. [22, p. 6] mentioned that residents should have to get out of their private room in order to engage with others.

Sometimes the communal space can also give rise to distraction and confusion though. Klaassens and Meijering [25] stated that residents may be easily distracted by other residents and by staff in communal rooms. Hauge and Heggen [23, p. 464] came up with set of findings that relate to the living room of nursing homes. Interior symbols in the living room, such as family photographs, carpets, and tablecloths, should be clear and consistent and make the room feel like a living room instead of a waiting room. A similar description of nursing homes not feeling like home was also given by Carboni [19, p. 35]. The living room was a difficult setting for engaging in private conversation with ten to twelve listeners sitting around all the time. Maintaining a certain degree of privacy, therefore, required the residents to leave the living room and go into their own rooms or the corridor. The same conclusion was drawn by van Zadelhoff et al. [31, p. 2494], who found that, to some residents, it is important to have the possibility to retire to one's room or withdraw.

Some other aspects of the public space were found to be important. Robinson et al. [29, p. 502] stressed the need for a place to walk. The lack of such opportunities could lead to increased physical deterioration. An ideal design put forward by one participant in the study by Fleming et al. [22] would be a circle so that people would not arrive at a dead end. van Hoof et al. [16] further added that it is important that residents are able to reach all parts of a space from a wheelchair. Fleming et al. [22] found that wide corridors and wide doorways were seen as vital for easy access for those with wheelchairs.

#### 3.3.3. Personal Belongings

Personal belongings seem essential elements in the development and maintenance of a sense of home [2, 15, 16, 19, 22, 23, 25, 30]. Creating attachment to place consisted of two dimensions: nesting and being in charge. Personal belongings help to create attachment to place in terms of in nesting and being in charge. Personalizing the environment, making room for personal belongings with furniture, and memorabilia can transform a private room to a place of recognition and familiarity that symbolized and strengthened one's self-identity, distilled from a lifetime of memories, experiences, and meanings attached to them [21, p. 1002]. Lewinson et al. [26, p. 750] stated that being able to bring personal histories into the space through beloved items and cherished furniture may contribute to feeling home. Many photographs contained items with emotional connections to previous homes. Jonsson et al. [24] found that certain pieces of furniture in their private rooms were only meant to accommodate significant others such as family or guests and therefore considered important. In addition, individual and unique pieces of furniture were a source of delight and were able to evoke memories [24].

van Hoof et al. [16, 30] researched the importance of personal possessions and found that there is a wide array of pieces of furniture or personal belongings that are present in the rooms of the residents. Small items such as drawings of grandchildren meant a lot to the participants, because they represented memories of loved ones [30, p. 6]. Among the items that were deemed most important were pictures, paintings, and pieces of furniture [16]. Personal belongings reflect the emotional value they bring to the individual. In addition, paintings have a particular decorative value and make the room more homelike. Some of the pieces of furniture were brought along at the time of admission for practical reasons, such as lift chairs. Often, there is an emotional value attached to the pieces of furniture, because they remind people of their former home or because they were purchased together with a partner [16]. Also, residents value the freedom in the choice and positioning of furniture in order to make the room cozier. To some of the residents, the personal belongings do not (yet) contribute to a sense of home, because the new living situation is rather overwhelming [16].

#### 3.3.4. Technology

The small category of technology describes the facilitating role that modern (communication) technologies can play in relation to improving the ease of life and being in charge and, in an indirect manner, could facilitate a sense of home. According to the findings by de Veer and Kerkstra [20], easy access to one's personal (i.e., patient) data contributed to feeling at home, and technology may play a role in this procedure. According to the study by van Dijck-Heinen et al. [2], technological solutions, which are already used by the participants, are of a different league compared to assistive technologies used by care professionals. These technological solutions are necessary for mobility or to ask for help after a fall incident. Some technologies which were already in use are considered to be acceptable, whereas future developments were approached with skepticism. Fleming et al. [22] stated that there is a need to approach the use of technology, so that it is understandable and acceptable to the person regardless of one's cognitive ability. In the field study by van Hoof et al. [16], the researchers found that the television sets were considered the most valued item in the private room. It provided the residents with a link to the outside world and distraction.

#### 3.3.5. Look and Feel

The factors constituting “look and feel” relate to architecture, interior design, and the general maintenance. In the study by Robinson et al. [29] and Fleming et al. [22], the authors stated that, in order to be counted as home, the facility needed to both look and feel like home or have a homely feel. The building should be “homey,” more organized, and more welcoming of family members [29].

Satisfaction was negatively influenced by concerns about the physical design, as well as cleanliness. Cleaning tools and products, kept inside the private room, had a certain meaning in the study by van Hoof et al. [30]; namely, there was a sense that the room was kept clean and tidy. In addition, flowers may contribute to a homely atmosphere [30]. The appreciation for cleanliness and decorations was also acknowledged by Lewinson et al. [26, p. 750].

The decor, color, warmth, and light of the facility were also important to residents in the study by Cooney [15, p. 194]. A homelike environment may also be created through putting residents' artwork on display. Participants did not report the same ease or contentment in settings that were not “homely” [15, p. 194]. van Dijck-Heinen et al. [2] further stated that a sense of home connected with warmth and coziness. Some of the residents mentioned daylight access, color, and a “fresh” appearance without smelly odors as being important. A hospital-like environment is something all residents participating in the study wanted to avoid. Furthermore, buildings with long corridors and “nooks and crannies” were deemed unsafe.

Fleming et al. [22] also stressed the engagement with the senses, by mentioning ventilation and a window nearby as well as music in order to provide comfort right at the end of life. Some residents may require an environment that is free of noise or free of excessive visual stimuli.

Finally, Jonsson et al. [24] stated that a physical environment that makes activities accessible and promotes pleasure and stimulation of all the senses with lighting and support of visual perception is especially important for those with limited mobility. Also, the physical comfort was a major aspect stated by the residents in the shared rooms. Chairs that did not fit the body shape were a major problem. A homelike appearance in the shared rooms was preferred to encourage a sense of home for the residents. Descriptions of furniture-related factors with homelike characteristics included an old style and wood as a material. Institutional environments with neutralized, single hue interiors without contrasts and natural lacquered wood furniture were not desirable.

#### 3.3.6. Outdoors and Location

The outdoor environment constitutes the outdoor space belonging to the own premises and the neighborhood at large. In the study by de Veer and Kerkstra [20, p. 432], there was a significant relationship with the degree of urbanization. Nursing homes located in large or very large cities had a higher proportion of residents not feeling at home in the nursing home. Nursing homes located in the own home town or the old neighborhood were preferred [2]. The presence of loved ones and children plays a role in this preference. Inaccessibility or loss of familiar places that provided the memories of past experiences is considered a negative factor in developing a sense of home [19]. Residents may also have a desire for going to shops and stores in the direct neighborhood for making small purchases [30].

Robinson et al. [29] provided an example of the important role of the outdoor environment, as participants expressed their concerns about the lack of trees and walkways outdoors. For them, “it was home-like indoors but not so outdoors” [29, p. 500]. Landscaping should be done with care. In the study by Robinson et al. [29], solid fencing that blocked a view was a reason for dissatisfaction. Many family members expressed dissatisfaction with the lack of connection to nature and the absence of a garden. In the study by van Hoof et al. [30], the green environment, in which the nursing home was located, was appreciated. This did not mean that all residents could appreciate the landscape by going outside, but in such cases the view of nature was considered to be attractive and pretty. Using outside spaces to facilitate engagement with the senses was seen as very important by caregivers [22]. Residents expressed a desire to go outside the facility more often in the study by Klaassens and Meijering [25]. Having a view in itself was also important to residents. Residents did not make a distinction between the types of view from the room [30]. Any type of view was appreciated, whether it was a park, traffic, a playground with children, a lively street, or a building [2, 30]. Residents value balconies and the views from the room [16].

## 4. Discussion

### 4.1. Main Findings

This is the first systematic review to identify factors that influence the sense of home of nursing home residents from their own perspective. Seventeen articles based on qualitative, quantitative, or mixed methods were identified. The majority of the data was based on qualitative research investigating factors of the sense of home among nursing home residents. Results show that the sense of home is influenced by 15 factors, divided into three themes: (1) psychological factors (including the sense of acknowledgement, preservation of one's habits and values, autonomy and control, and coping); (2) social factors (including relationships and interaction with staff, residents, family and friends, and pets) and activities; and (3) the built environment (including the private space and the (quasi-)public space, personal belongings, technology, the look and feel, and the outdoors and location) (Figure 2). Factors that were mentioned most often in the literature were the interaction with staff, autonomy and control, activities, the spaces, and role of personal belongings.

The three themes identified in this study were consistent with the results of previous work by Oswald and Wahl [32], who categorized factors constituting the sense of home into the following three groups: physical, social, and individual factors. These themes also emerged in research by Felix et al. [33] on the everyday lived experience of the house in The Netherlands, which was conducted with community-dwelling older people. The results also support earlier findings by Percival [34] on the uses and meanings of domestic spaces in the daily lives of older people in the United Kingdom. In this British study, older people require adequate, accessible, and personalized domestic spaces in order to facilitate three objectives, namely, routines, responsibilities, and reflection. The study by Róin [35] found that home as a concept has different meanings for different older people, depending on various conditions. Groger [36] found that the respondents' ability to consider a nursing home a “home” was related primarily to the criteria they used to define “home.” Other indicators that emerged from her study included the circumstances of nursing home placement, previous experience with nursing homes, and the degree of continuity achieved after placement. It seems that older people residing in the community and in nursing homes broadly have the same needs. However, they may face different boundary conditions in the extent to which they are autonomous in their choices and actions. In this view, coping styles and being able to deal with living in a nursing home in general are important. Residents who are not open to the new situation or environment may never be able to feel at home in a nursing home [14]. Mortenson et al. [37] studied the impact novel surveillance technologies would have on the everyday experience of home in community-dwelling older people. They concluded that new technologies may alter the perceptions of home as surveillance technologies increase the permeability of the home by extending the power of observation into what was previously regarded as an intimate and private space. In the near future, novel care technologies may also pose an unknown influence to the development of a sense of home in the nursing home, which deserves attention in future studies and practice.

The main challenge for future work is how to move from specifying themes to the identification and development of feasible and replicable evidence-based interventions that contribute to the sense of home. Based on the themes and factors identified in this study, scales and questionnaires could be developed as a first step to objectively measure the aspects of a sense of home that nursing home residents experience. No studies, to our knowledge, have investigated these aspects in residents of nursing homes on a large scale. Regarding the gathering of objective evidence of at-homeness, there is a need for conducting larger, longitudinal studies using a reliable and valid measure of at-homeness such as the Experience of Home Scale [38]. We need to examine predictors and sequelae of at-homeness in both small group homes that incorporate architectural qualities of home as well as social and care-related interventions [27]. For instance, the U.S. model of the so-called Green House nursing homes [39], which are created to be a “real home,” yet lacks clarity on the mediating role of the perceived or lived experience of at-homeness on the outcomes. In many Dutch small group homes, homelikeness is propagated, whereas the sense of home may be more fitting and appropriate construct to consider. As the sense of home encompasses social, psychological, and architectural aspects, it remains to be determined whether these aspects should be addressed, in terms of interventions, separately or holistically. Preliminary work shows that certain aspects of Dutch small group homes are successful in providing a homelike atmosphere, such as privacy, own choice, and emphasizing autonomy [40]. Nursing staff and healthcare managers can create a sense of personal freedom in which residents can decorate their rooms according to personal preferences. In order to measure the impact of improvement efforts, there are more tools available within the domain of nursing sciences compared to the domain of architectural studies to measure effects. Future research should address all these aspects and measure their effectiveness. As for coping styles, this is an area not studied extensively in the context of nursing home residents [41, 42].

The factors identified in this study relate to the sense of home of nursing home residents. However, this review also elaborated further on aspects that have an indirect relationship to the sense of home. These factors were taken into account for two reasons. First, the additional information in this review provides important guidance for practice. An example of this is the text about family involvement and large apartments to receive visitors. Second, these additional aspects possibly mediate associations with the sense of some, for example, the need to feel known and valued as an individual. Some studies describe that identity of residents is the strongest when their sense of their own value and uniqueness and other people's actions that communicated their worth matched. The concept of dignity seems very relevant here, and it is not only just a part of a psychological theme, but also interactional and part of a larger social theme. All kinds of engagement identified in this study, for instance, the engagement with one's senses and spiritual engagement, highlight the interaction within and across themes.

Some of the factors identified in this study, for instance, the sense of acknowledgement, describe that the identity of residents was the strongest when their sense of their own value and uniqueness and other people's actions that communicated their worth matched. The concept of dignity seems very relevant here, and it is not only just a part of a psychological theme, but also interactional and part of a larger social theme. All kinds of engagement identified in this study, for instance, the engagement with one's senses and spiritual engagement, highlight the interaction within and across themes.

Apart from these interrelationships, there are also tensions between factors. There may be tensions between resident preferences and family preferences, in relation to autonomy and control on the one hand and continuity of family relationships on the other hand [31]. There may also be tensions that emerge when health and functional disability limit the ability to engage in behaviors, activities, rituals, or social interactions that limit the ability to preserve continuation of old life. Not all nursing home organizations seem to be able to come up with adequate solutions in order to try to create a sense of home. In future studies, the prioritization of needs should be studied in order to gain a better understanding of such tensions and how these tensions can be resolved.

### 4.2. Strengths and Limitations

This review's strengths lie in its extensive search strategy, covering databases in the fields of social sciences, health care, and architecture. This systematic and multidisciplinary approach is also reflected in the extraction of factors from qualitative research, which was done by two independent reviewers from different backgrounds (psychology and engineering) and verified by several researchers with a background in medicine, health studies, architecture, and applied gerontology. Another strength is the inclusion of all types of available evidence, regardless of the type of research method (qualitative, quantitative, or mixed methods). The qualitative data included in this study was rich and yielded many factors and illustrative quotes.

Some limitations have to be acknowledged. First, moderating or mediating relationships between factors were not investigated in this review due to lack of available data. However, this review is one of the few studies that investigated the sense of home in a holistic manner, including both the social and care environments, as well as the architecture and interior design of the nursing home. Another limitation to this study is that studies were not appraised in terms of their methodological strength. Results of the included studies were treated equally. Finally, the studies included in this review gathered data from Western countries from three continents (North America, Australasia, and Europe). The results may reflect the sense of home as it is experiences in Western contexts, and the findings could, therefore, be different if studies from Asia would have been available.

### 4.3. Implications for Practice

This review has identified 15 major and minor factors influencing the sense of home of nursing home residents. Most findings can be directly implemented into practice. Care professionals, for instance, can pay attention to their actions and attitudes towards the sense of home of nursing home residents. In addition, managers and policy makers are offered practical guidance on how to improve facility-related aspects that influence the sense of home, for example, the built environment. The findings of this study need to be viewed in the context of policy and practice. In every country, policies related to nursing home administration and financing, as well as care practices, differ, and so do national building codes, methods of construction, and architectural preferences. Nevertheless, this review forms a basis for future studies which should help identify the exact needs of residents, within a national context or within certain financial constraints.

*Attitudes and Social Interaction*. Nursing staff could start engaging with the residents and stimulate them in having social interaction with others and taking parts in activities which are meaningful and make the best use of remaining capabilities. Relatives should be welcomed to give a helping hand and should also feel at home to some degree. This requires a different set of skills of nursing staff other than being qualified to provide care, for instance, empathetic, social, and communicative competences. 

*The Built Environment*. The general attitude of a facility should be welcoming, not only on a social engagement level, but also in relation to being free in redesigning and decorating the private rooms and even the communal living rooms or corridors. Being able to decorate a room enables residents to turn the nursing home space into a private room which has a connection to the previous life. Residents, who wish to decorate their room, paint it in a different color, or put up artwork, should not be confronted with strict rules but should be invited to make changes and engage in a dialogue (supported by a loved one) to mutually explore the boundaries of their actions. Architects should consider such needs when designing, retrofitting, or transforming nursing homes, for instance, by providing sufficient space for personal belongings. 

*The Outdoor Environment*. Gardens and neighborhoods should be available to residents who are still able to enjoy a fitting degree of freedom. They form an integrated part of life in a nursing home. Despite these recommendations, one needs to be aware that the development of a sense of home depends on a large number of factors that may vary for each individual. One solution to raise awareness among professional caregivers could be the provision of factsheets containing hints for improving the factors identified in this study. Education and training may be other instruments to improve the living conditions in nursing homes.

## 5. Conclusions and Recommendations

This study has shown that the sense of home of nursing home residents is influenced by a multitude of psychological and social factors, as well as the built environment of nursing homes. Further research is needed to determine if and how the factors in this review are interrelated, if there are differences in perspectives between the various stakeholders involved, and how the factors can be implemented in practice as viable solutions to improve the sense of home. The built environment is an import theme which should be considered when trying to improve the sense of home of nursing home residents. These environmental factors are not always addressed in practice, as they are not considered a core item in the provision of health care. Therefore, it should be stimulated to have architects, designers, and care professionals work together in the creation of optimal designs of nursing home environments. In relationship to the social and psychological factors, a person-centered approach in nursing home care seems important, and this person-centeredness should also include the relatives of the residents. The quality of the interaction with care staff and the activities that are being conducted deserve more attention in daily practice and offer a means for improving the sense of home. Finally, we recommend addressing the interaction between the psychological, social, and environmental components of living in a nursing home in a holistic manner. To date, there is insufficient evidence on how to stimulate this interaction. In the end, the sense of home is centered on balancing these three aspects. In practice, the emphasis is either on quality of care and interaction or on the quality of the built environment. A better way forward may lie in stimulating empathic care professionals in how to use the building and its interior design to the full advantage of their professional task and the residents.

## Figures and Tables

**Table 1:** Characteristics of the 17 reviewed articles.

Author (year) [reference]	Design and number of participants	Setting	Goal of study	Notes
Bland (2005) [12]	Interview (*n* = 1).	Nursing home, psychogeriatric ward in New Zealand.	To provide insight into how people in New Zealand feel at home in a nursing home.	

Carboni (1990) [19]	Hybrid model of field observations and theoretical analysis. Unstructured interviews (*n* = 2).	Nursing home with 120 beds in the USA.	To study if residents feel “at home” (and how this is defined for the participants) and if this has a specific meaning.	It is hard to differentiate between the field observations and theoretical analysis. It is not clear if any of the residents have a psychogeriatric background.

Cooney (2012) [15]	Unstructured interviews with residents (*n* = 61).	Public and private long-term care settings in Ireland.	To understand older people's perceptions of “being at home” in long-term care settings and the factors that influence these perceptions.	

de Veer and Kerkstra (2001) [20]	Quantitative study with 686 residents (with both mental and physical health problems) or significant others of residents.	36 nursing homes in the Netherlands.	To examine the determinants of “feeling at home” in nursing homes	

Falk et al. (2013) [21]	Qualitative study comprised of interviews (*n* = 25).	Several care institutions in Sweden.	To understand the strategies creating a sense of home.	

Fleming et al. (2015) [22]	Focus groups (FG) with family caregivers of people with dementia (FG1) and people with dementia and family caregivers of people with dementia (FG2) and practitioners caring for people with dementia nearing or at the end of their lives (FG3) (*n* = 4 + 9 + 5).	Focus groups were conducted among people suffering from dementia in three big cities in NSW, Australia.	To explore the views of people with dementia, family caregivers, and professionals on what aspects of the physical environment would be important to support a good quality of life to the very end.	There is no clear distinction if participants of focus groups 1 and 2 lived at home or in a nursing home. Focus group 3 included a care home manager, a dementia care educator, and a palliative care nurse researcher. Their work experience could be obtained at home or in the nursing home.

Hauge and Heggen (2008) [23]	Participant observation and interviewing (*n* = 24).	Two Norway based long-stay units with 12 residents in each unit.	To study how and to what extent the ideas of a nursing home as a home have been realized.	

Jonsson et al. (2014) [24]	Interviews (*n* = 12).	Three nursing homes in Stockholm, Sweden.	To explore the relationship between furniture and people.	

Klaassens and Meijering (2015) [25]	Observations, in-depth interviews, and diaries. Study includes both caregivers and residents. All 20 residents were observed. Interviews were held with 5 residents and 3 caregivers.	Korsakov ward of within larger nursing home in the Netherlands.	To gain insight into (1) the features of home and institution as experienced by residents and caregivers of a secured ward in a nursing home and (2) how interventions implemented on the ward can contribute to a more homelike environment.	

Lewinson et al. (2012) [26]	Qualitative, photovoice method (*n* = 10).	A large assisted living facility in Atlanta, GA, USA.	To explore home through residents' eyes.	

Molony et al. (2011) [27]	Mixed-methods study, *N* = 25 nursing home residents.	100-bed nursing home in USA. A descriptive, longitudinal mixed-methods design with four waves of repeated measures was used to achieve study aims. Interviews were conducted after residents made the decision to stay or move (baseline) and at 1, 3, and 6 months after the person relocated to the small-scale nursing home.	To examine trajectories of *at-homeness *over time in two groups of older adults who were given a choice to move to a small house nursing home or stay in their existing residence, a usual care nursing home to provide insight into the complex relationships between individual needs and desires, the LTC environment, at-homeness, and health.	The qualitative examples illustrate the individualized nature of at-homeness and the necessity of smooth integration of both the “social model” and the “medical model” of care.

Nakrem et al. (2013) [28]	Interview with 15 residents.	4 small-, medium-, and large-sized nursing homes in both urban and rural areas in Norway. The nursing homes had mixed populations according to medical diagnosis, physical and cognitive functioning, and age.	To study life in nursing homes in relationship to quality of care.	

Robinson et al. (2010) [29]	Interviews and focus groups with 29 family caregivers.	2 nursing homes in USA.	To study the meaning of home according to family of people with dementia.	

van Dijck-Heinen et al. (2014) [2]	Interviews and focus group session (*n* = 10, of which *n* = 4 permanent residents and *n* = 6 temporary residents).	One nursing home organization in the Netherlands.	To investigate the sense of home and its constituent factors among both permanent and temporary residents.	Study included 6 temporary residents (rehabilitation).

van Hoof et al. (2016) [16]	Interviews with 27 residents.	5 nursing homes in the Netherlands	To find out which personal possessions were most important and if they contribute to a sense of home.	

van Hoof et al. (2015) [30]	A photo production study. Interviews with 12 residents.	2 wards of 1 nursing home in the Netherlands for residents with physical limitations and gerontopsychiatric health problems.	To investigate which factors in the physical and social environment correlate with the sense of home of the residents and which environmental factors are most meaningful.	

van Zadelhoff et al. (2011) [31]	Observations and interviews with residents, their family, and nursing staff (*n* = 5 + 4 + 5).	Two group living units, located on the grounds of a traditional large-scale nursing in the Netherlands.	To investigate experiences of residents, their family caregivers, and nursing staff in group living homes for older people with dementia and their perception of the care process.	
